# Study on brittleness characteristics of deep shale: A case study of Lu211 well in the Luzhou block

**DOI:** 10.1371/journal.pone.0308359

**Published:** 2024-12-12

**Authors:** Bo Zeng, Guozhou Qiu, Liqing Chen, Yintong Guo, Shouyi Wang, Junchuan Gui

**Affiliations:** 1 Shale Gas Research Institute of PetroChina Southwest Oil & Gas Field Company, Chengdu, Sichuan, China; 2 Sichuan Key Laboratory of Shale Gas Evaluation and Exploitation, Chengdu, Sichuan, China; 3 State Key Laboratory of Geomechanics and Geotechnical Engineering, Institute of Rock and Soil Mechanics, Chinese Academy of Sciences, Wuhan, China; 4 School of Pipeline and Civil Engineering, China University of Petroleum (East China), Qingdao, China; Guizhou University, CHINA

## Abstract

Ensuring the sustainability of energy is pivotal for achieving a harmonious balance between environmental conservation and economic growth. The mechanical behavior of deep shale reservoir rocks is intricate, presenting challenges in ascertaining their brittleness characteristics. To address this, the study employed a suite of evaluation techniques, encompassing analyses of stress-strain curve attributes, energy dissipation patterns, and mineral composition profiles. The overarching goal was to delineate the variations in deep shale brittleness as a function of depth. The findings indicate a general trend of decreasing shale brittleness with increasing depth. However, the brittleness indices derived from the three distinct evaluation methods varied, with the mineral composition approach yielding the most scattered results. This disparity underscores the heterogeneity of deep shale, likely due to its varied diagenetic history compared to shallower formations. In response to these observations, the study leveraged the principle of weighted averaging to devise a composite brittleness evaluation method. This innovative approach not only integrates the effects of multiple influencing factors but also accounts for the differential impact and weight of each method on the overall brittleness assessment. By doing so, it offers a more nuanced and holistic understanding of shale brittleness. The paper’s exploration of deep shale’s brittleness characteristics contributes valuable insights for the exploration and development of deep shale reservoirs, enhancing the strategic and operational frameworks within the energy sector. This comprehensive evaluation method serves as a foundation for more informed decision-making, ensuring that energy extraction is conducted in a manner that is both economically viable and environmentally responsible.

## 1 Introduction

As the search for oil and gas continues to progress, and development technology and the increasing demand for oil and gas resources in production and life, the research focus of oil and gas exploration and development has gradually changed from conventional reservoirs to unconventional reservoirs, and unconventional oil and gas exploration and development has gradually become a focal point in the energy sector [1]. With the understanding of shallow reservoir becoming more and more full, the focus of exploration and development research has gradually expanded from shallow reservoir to deep reservoir. Shale reservoir is a typical multilayered unconventional reservoir. Shale gas has become a hot spot in oil and gas exploration and development because of its wide distribution and large resources. The breakthrough of shale gas extraction technology in the United States has transformed the country from a natural gas importer to a natural gas exporter, fundamentally changing the energy structure of the United States [2].

Brittleness refers to the characteristic of rock to shatter and disintegrate under the influence of stress, and this property is directly related to the practical application of geological engineering and rock mechanics [3]. Through the comprehensive evaluation of rock brittleness, the formation activity can be effectively predicted. The key factors of brittleness evaluation include the mineral composition of the rock, the distribution of cracks, and the stress state. In brittle rocks, the stress is often able to rapidly trigger crack propagation and breakage, while in ductile rocks, the rock may be more prone to plastic deformation than fracture. Consequently, precisely assessing the brittleness of rock is instrumental in devising suitable geotechnical designs and mining tactics, thereby enhancing the dependability and security of the project [4].

Due to the particularity of deep shale, there is no unified standard for brittleness evaluation of shale rock [5]. The difficulty in brittleness evaluation of shale rock is mainly due to the complex microstructure and mineral components of shale rock, and the existence of a large number of folds, faults and other irregular structures and geological structures in shale reservoir, resulting in non-uniform distribution of stress field. The stress states in different directions and depths are significantly different in shale reservoirs [6]. The followings are some of the main reasons leading to difficulties in shale brittleness evaluation: **a. Heterogeneity of microstructure**: The microstructure of shale is complex, and micro-fissures of different sizes are often distributed, which makes it more difficult to predict and describe the fracture and fracture behavior of shale [7]. **b. Stress sensitivity:** Shale rock is highly sensitive to stress, and its brittleness behavior may change to different degrees under different stress conditions. Therefore, brittleness evaluation needs to consider the changes under different stress states, which increases the difficulty of evaluation [8]. **c. Directional differences:** The brittle behavior of shales usually varies significantly in different directions. This is because micro-fractures and bedding structures in shale are not evenly distributed in different directions, resulting in spatial heterogeneity of brittleness behavior [9]. **d. Influence of chemical composition:** chemical factors such as organic matter content, mineral composition and hydration degree in shale will affect its brittleness behavior [10]. The complex interaction of these factors increases the difficulty of brittleness evaluation [11].

The brittle characteristics of rock were studied mainly from three aspects: triaxial test, acoustic wave test and mineral composition. Triaxial test is one of the most widely used methods. In triaxial test, brittleness characteristics of rocks can be studied from many aspects [12]. For example, brittleness index of rocks can be determined by analyzing energy changes in elastic and plastic stages of curves [13], and brittleness characteristics can be evaluated by analyzing stress-strain curves from the aspect of deformation characteristics. The acoustic wave test is mainly aimed at analyzing the brittleness characteristics of rocks in view of the fast propagation speed of brittle minerals in rocks. The brittleness characteristics of rocks can be inferred by measuring parameters such as the propagation speed, amplitude and attenuation of sound waves [14]. Moreover, through sound waves, some microscopic results in the rock can be monitored, such as micro-cracks, etc. The arrangement of micro-cracks will make the rock show anisotropic mechanical characteristics, which will affect the brittleness characteristics of the rock [15]. The brittleness of rock is closely related to its mineral composition, which can affect the hardness, fracture, deformation and other mechanical properties of rock, thus affecting the overall brittleness of rock [16]. The hardness of the minerals in the rock varies greatly, and the higher hardness of the minerals (such as quartz, pyroxene) will make the rock harder, but also more prone to brittle fracture [17]; Different minerals have different fracture characteristics, such as cleavage and fracture morphology, and the fracture plane formed by some minerals in the rock may cause the rock to be more prone to fracture when stressed.

In conclusion, the challenge in evaluating the brittleness of shale primarily stems from its intricate microstructure, the interplay of various factors, and its inherent heterogeneity. This paper studies the brittleness characteristics of shale in the same well with the change of depth, taking each small layer of Lu211 well as the research object. Triaxial compression test with confining pressure of 110MPa was designed and carried out, and the stress-strain characteristic curve was obtained, based on which the brittleness index (stress strain method) was evaluated. Based on the stress-strain curve of triaxial test and the principle of energy release, the brittleness index of deep shale is analyzed from the perspective of energy. The mineral components of different small shale layers were obtained by XRD test, and the brittleness index of deep shale was evaluated by analyzing the content of brittle minerals. Based on the principle of weighted average, a brittleness evaluation method considering many factors was proposed.

## 2 Test procedure

### 2.1 Deep shale core acquisition and processing

#### 2.1.1 Underground core collection

The underground core is taken from Luzhou Block, and the specific sampling information is shown in Table 1.

**Table 1:** Coring information.

Well	Position	Depth/m
Lu211	Longyi-_1_^4^	4900.92–4901.16
	Longyi-_1_^3^	4908.02–4908.30
	Longyi-_1_^2^	4917.64–4917.86
	Longyi-_1_^1^	4923.87–4924.05
	Wufeng	4929.39–4929.62
	Baota	4933.32–4933.54

#### 2.1.2 Sample processing

**Fig 1:**
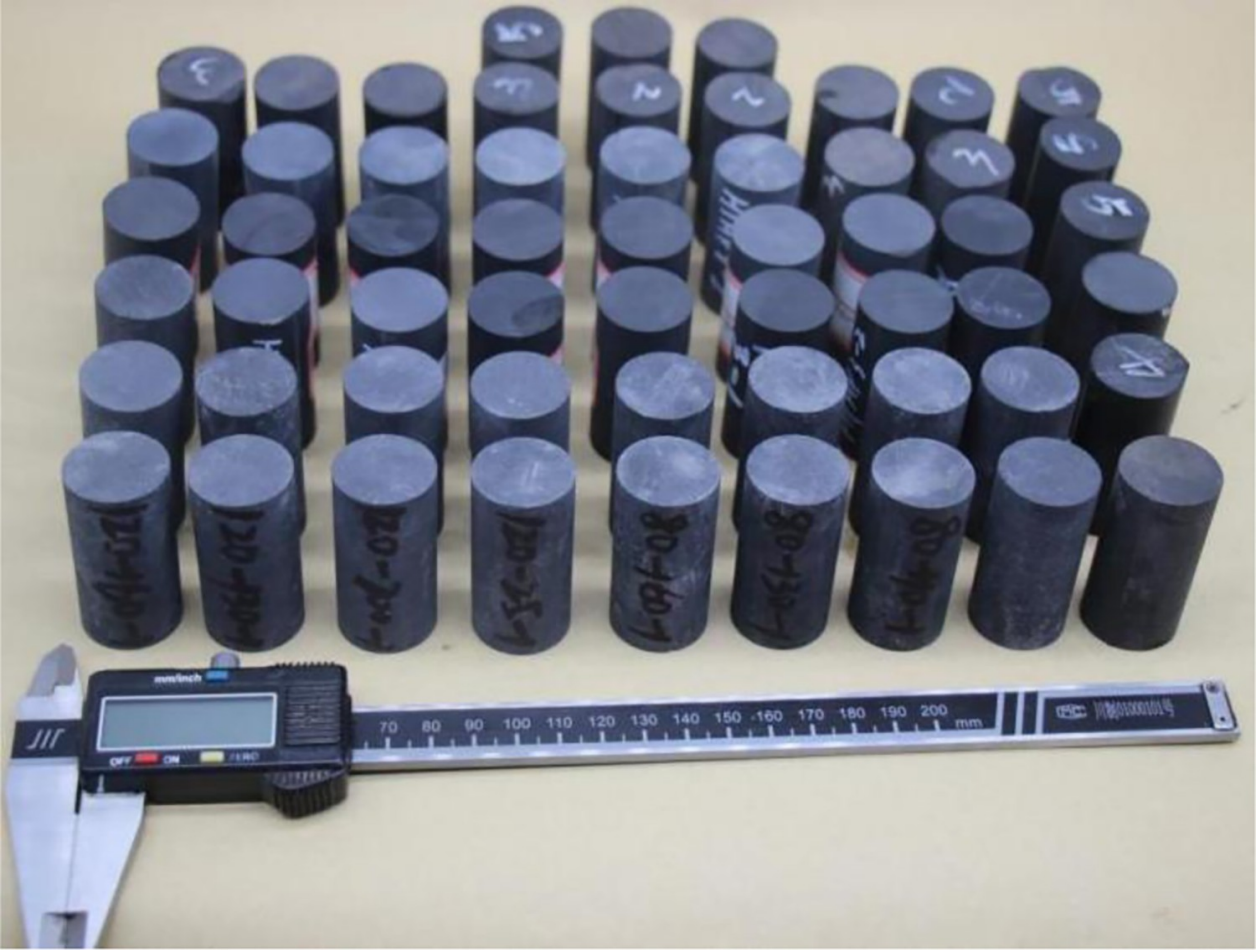
Part of the core used for testing.

Water has a great impact on the mechanical properties of deep shale, so the method of waterless wireline cutting was used to prepare samples. The two ends of the cylindrical sample were ground into a plane with a non-parallel degree of less than 0.02mm, and a rock core with a diameter of *Ф*25×50 mm was finally obtained, as shown in Fig 1.

### 2.2 Test instrument and research method

**Fig 2:**
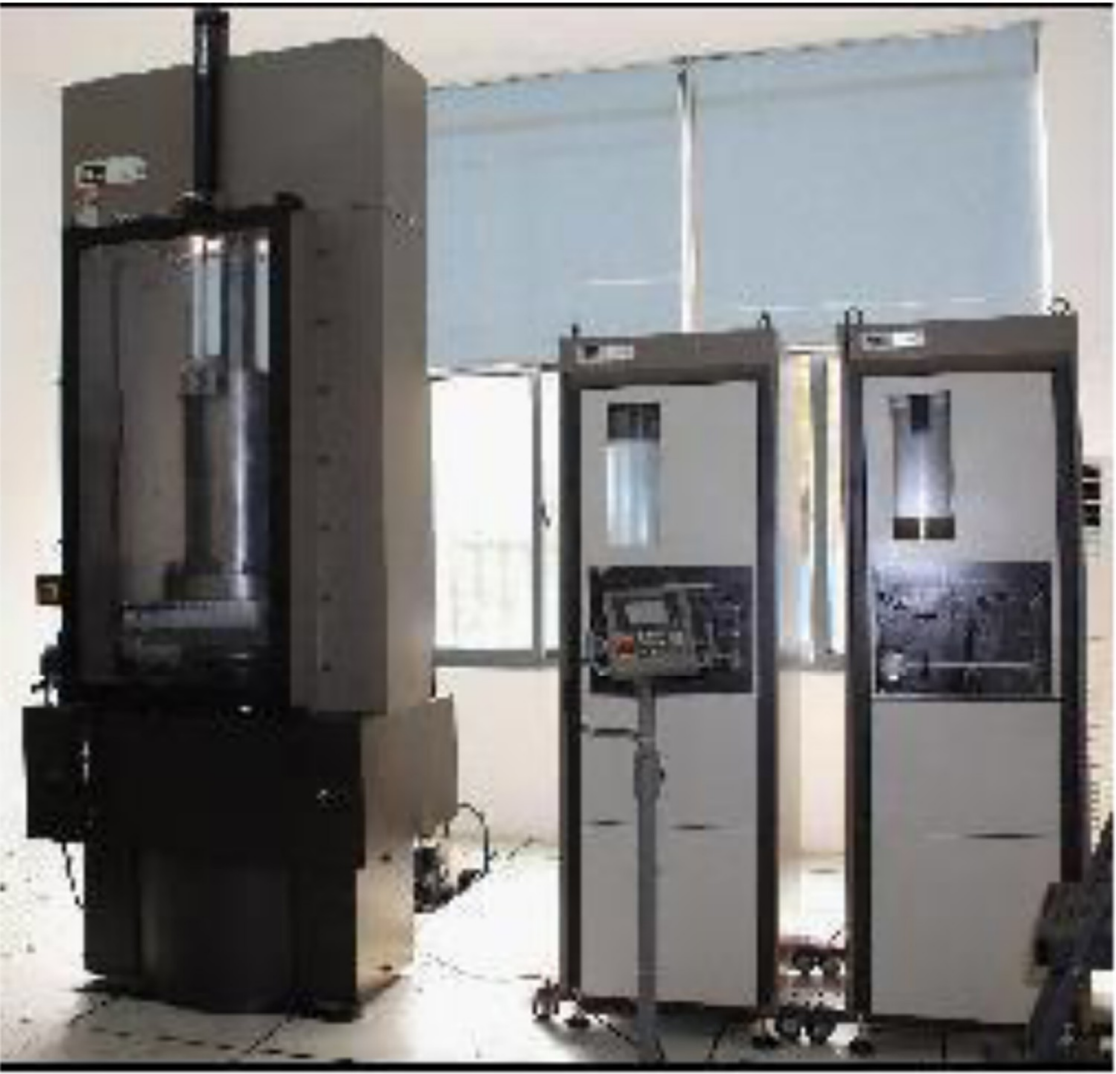
MTS815 flex test GT.

The equipment used in this Test is the MTS rock mechanics test system, as shown in Fig 2. Primarily employed for standard mechanical testing of rock, concrete, and similar materials.

**Fig 3:**
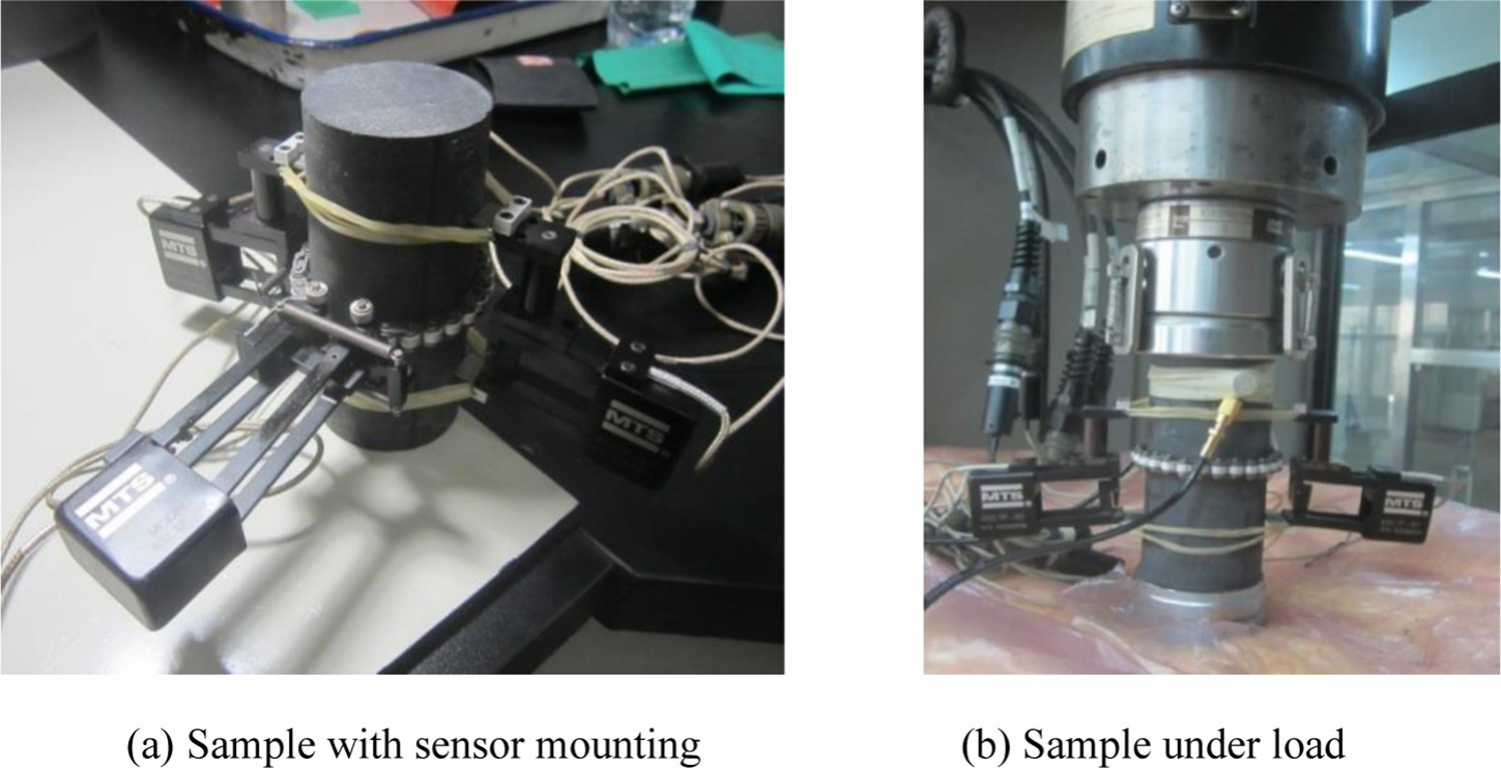
Flow chart of installation. (a) Sample with sensor mounting, (b) Sample under load.

Before the test, the polytetrafluoroethylene gasket is placed between the two ends of the rock sample and the indenter of the testing machine to more effectively isolate the noise and reduce the end friction. An axial extensometer and a circumferential extensometer (installation process shown in Fig 3) were installed on the sample to measure axial and radial deformation.

## 3 Analysis and outcomes of tests

### 3.1 Stress-strain method

**Fig 4:**
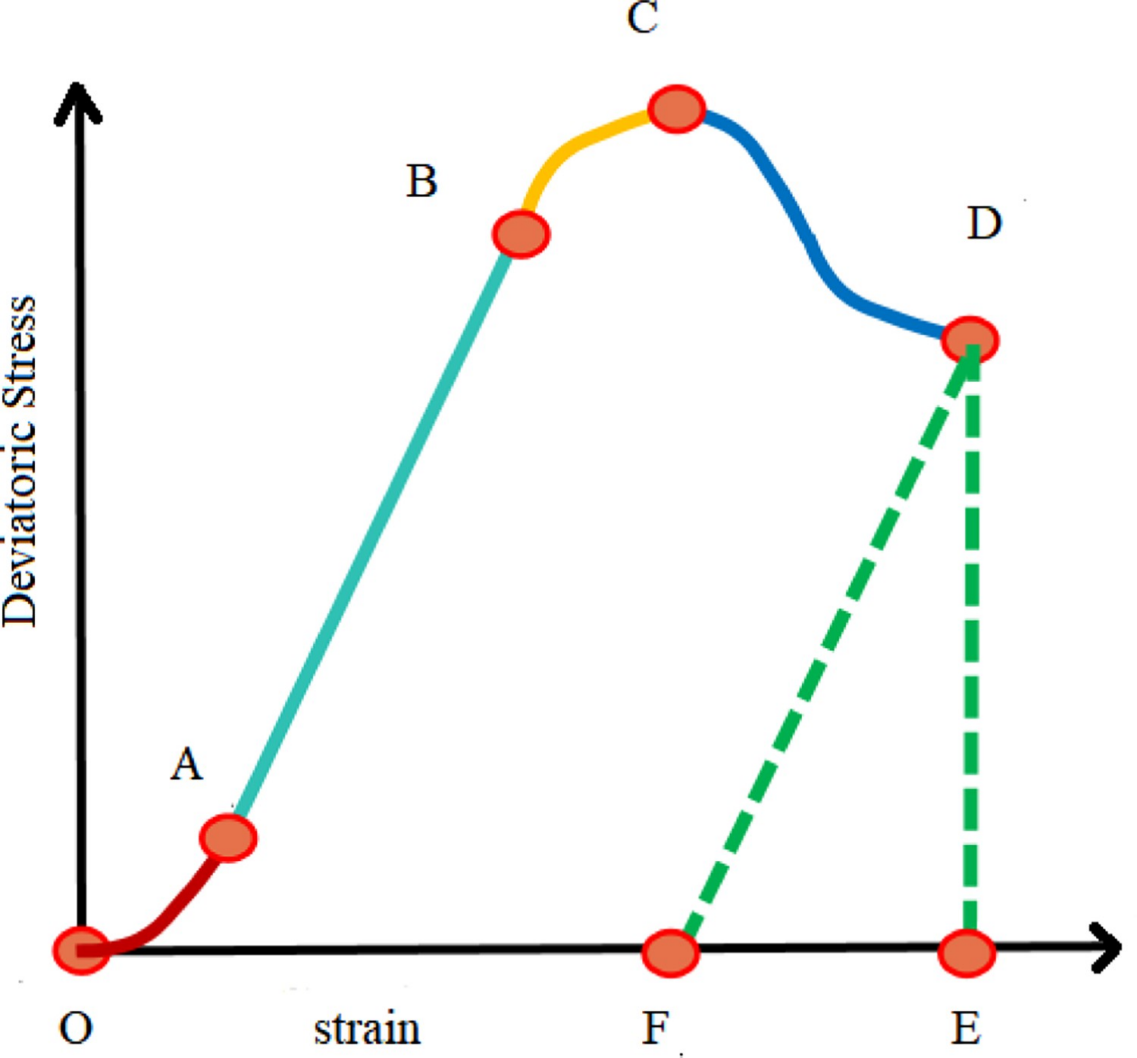
Method for evaluating brittleness based on characteristics of stress-strain curves.

According to Hucka [18], The brittleness of rocks can be evaluated by stress-strain curve characteristics, as shown in Fig 4. Due to the large number of pores and micro-cracks in the interior of the rock, closure occurs when the axial force is applied, that is, the OA section is the compaction stage. This is followed by the elastic deformation stage, during which the axial stress should become proportional to the axial direction, as shown in section AB in the figure. BC stage is the plastic deformation stage, in this stage, the rock began to appear damage and large deformation. When the rock reaches point C, it loses its bearing capacity and fails, and stage CD is the stage of residual deformation.

Brittleness is defined as the ratio of the elastic strain to the total strain value. To wit:

$$BI_{1}=\frac{\mathrm{Reversible}\;\mathrm{strain}}{\mathrm{Total}\;\mathrm{strain}}=\frac{EF}{OE}$$ 3-1

**Fig 5:**
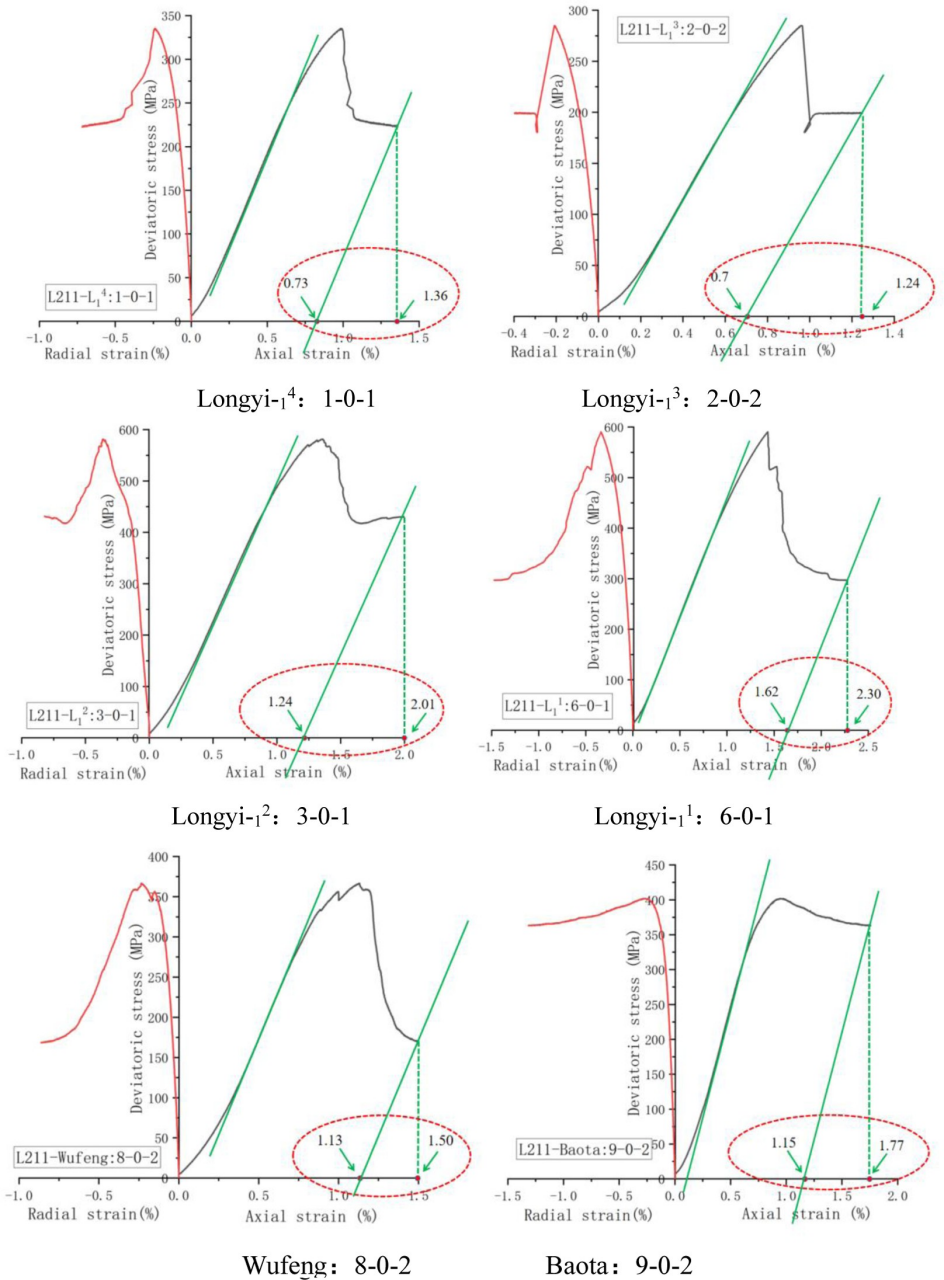
Brittleness evaluation of Lu211 well (stress strain method). Longyi-_1_^4^: 1-0-1, Longyi-_1_^3^: 2-0-2, Longyi-_1_^2^: 3-0-1, Longyi-_1_^1^: 6-0-1, Wufeng: 8-0-2, Baota: 9-0-2.

Mark the stress-strain curve of the triaxial test according to the above formula, and the results were shown in Fig 5.

**Fig 6:**
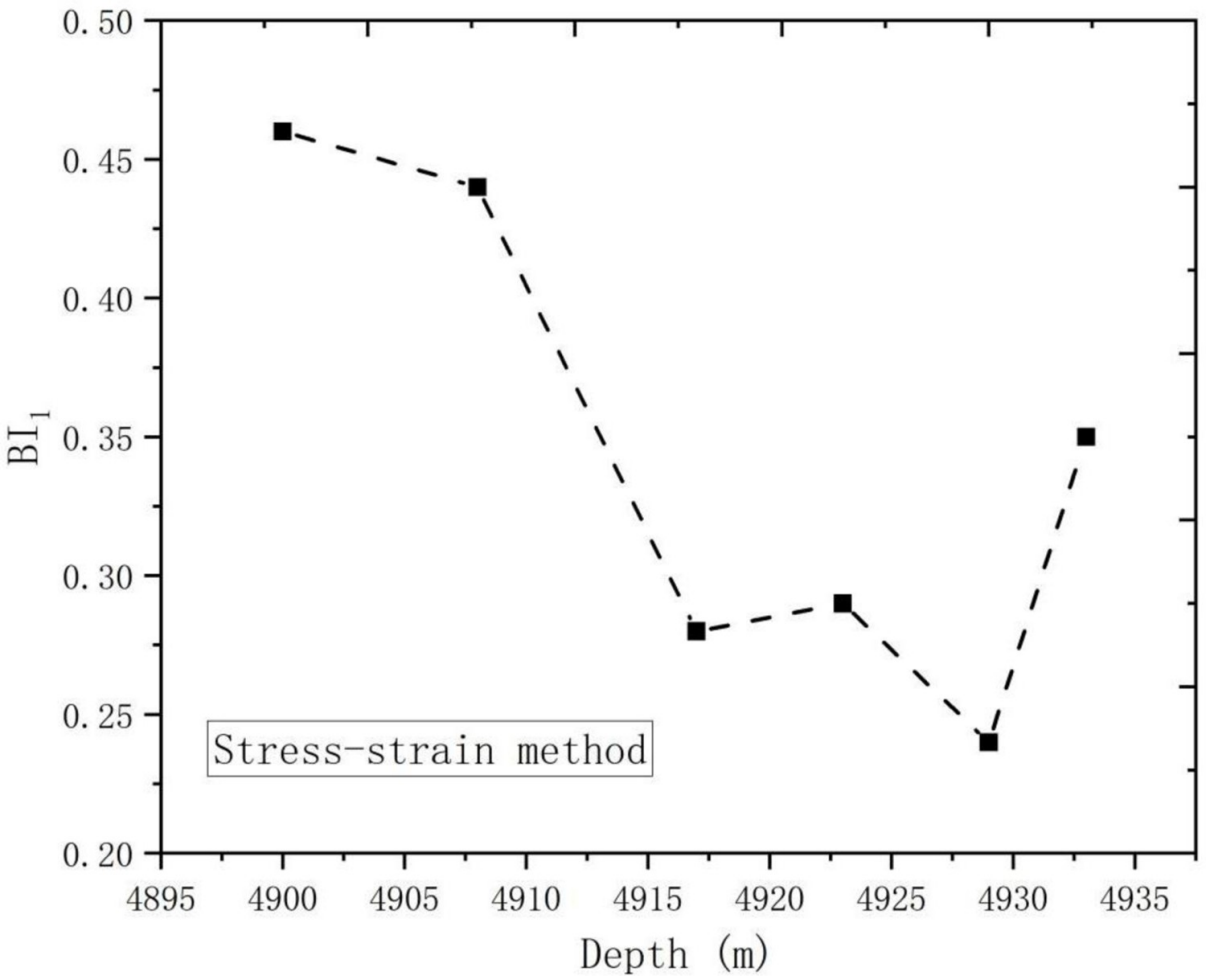
Brittleness index trend graph with depth (stress strain method).

According to Fig 6, the obtained test results were shown in Table 2.

**Table 2:** Rock mechanics parameters of Lu211 well under triaxial compression.

Well	Position	Depth/m	Number	Confining pressure/MPa	EF(%)	OE(%)	BI_1_
Lu211	Longyi-_1_^4^	4900.92–4901.16	1-0-1	110	0.63	1.36	0.46
	Longyi-_1_^3^	4908.02–4908.30	2-0-2		0.54	1.24	0.44
	Longyi-_1_^2^	4917.64–4917.86	3-0-1		0.77	2.01	0.28
	Longyi-_1_^1^	4923.87–4924.05	6-0-1		0.68	2.30	0.29
	Wufeng	4929.39–4929.62	8-0-2		0.37	1.50	0.24
	Baota	4933.32–4933.54	9-0-2		0.62	1.77	0.35

It is evident from Fig 1, the brittleness index of deep shale decreased with the increase of depth. On the one hand, the stress state of shale increased with the increase of depth, the porosity of rock is lower, the micro-cracks of rock are closed, and the bedding is closer, so the rock is more dense, which will increase the tensile strength of shale, and then reduce the brittleness of shale. However, the brittleness index in Baota formation increases, which may be due to the sudden increase in the content of the brittle mineral calcite in the mineral components, as shown in Table 4, indicating that the mineral components in the shale undergo phase changes with the increase of depth, and the calcite in Baota shale is about 10 times that in other formations. It can be seen from the stress-strain curve that with the increase of brittle minerals, the slope of the elastic stage of the rock is higher, the elastic modulus is larger, but the post-peak modulus is smaller, so the final calculated brittleness index is larger.

### 3.2 Energy dissipation method

Tarasov B [19] considered the energy accumulated after the peak of the stress-strain curve as a key factor in evaluating rock brittleness.

**Fig 7:**
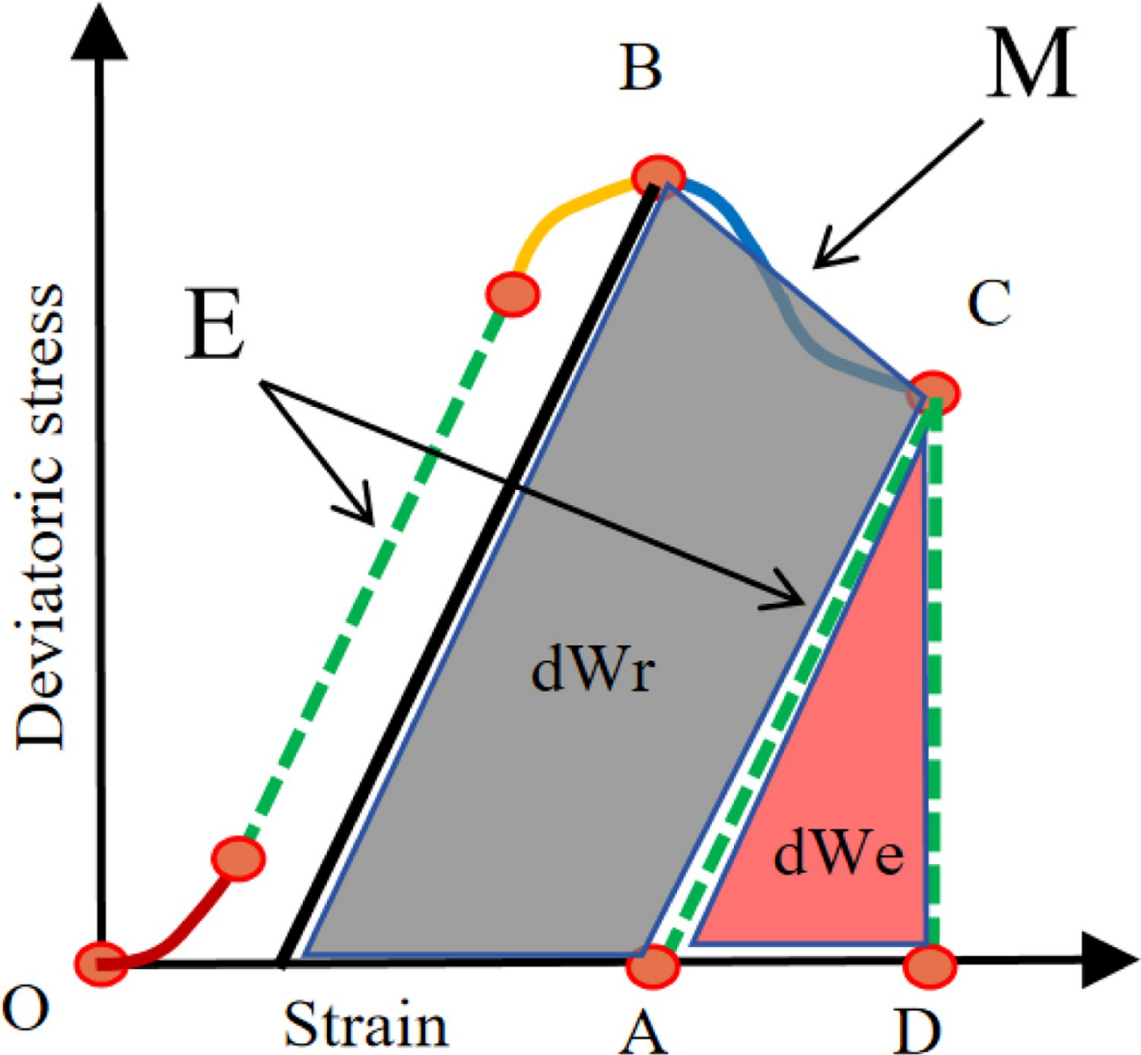
Brittleness evaluation based on energy dissipation method.

Fig 7 shows an overview of brittleness evaluation by energy dissipation method. The area dWe of the red triangle in the figure describes the elastic energy of the sample, and the deformation of the rock sample in this stage recovers after the stress is relieved. The gray area dWr represents the energy released by rock fracture and the energy accumulated during elastic deformation.

The brittleness index reflects the material’s inherent instability at failure. To simplify the estimation of the elastic energy dWe extracted from the sample during post-peak failure between points B and C (highlighted in red on the right), it is assumed that the elastic modulus E = dσ/dε remains constant at both points [20].


$$dW_{e}=\frac{\sigma_{B}^{2}-\sigma_{C}^{2}}{2E}$$ 3-2

The graph indicates that the fracture energy dWr after the peak is calculated by adding the extracted elastic energy dWe to the additional energy represented by the gray area.


$$dW_{a}=\frac{\sigma_{B}^{2}-\sigma_{C}^{2}}{2M}$$ 3-3

The post-peak rupture energy dWr can be expressed by the following equation:

$$dW_{r}=dW_{e}-dW_{a}=\frac{\left(\sigma_{B}^{2}-\sigma_{C}^{2})(M-E\right)}{2EM}$$ 3-4

So

$$BI_{2}=\frac{dW_{r}}{dW_{e}}=\frac{M-E}{M}$$ 3-5

**Fig 8:**
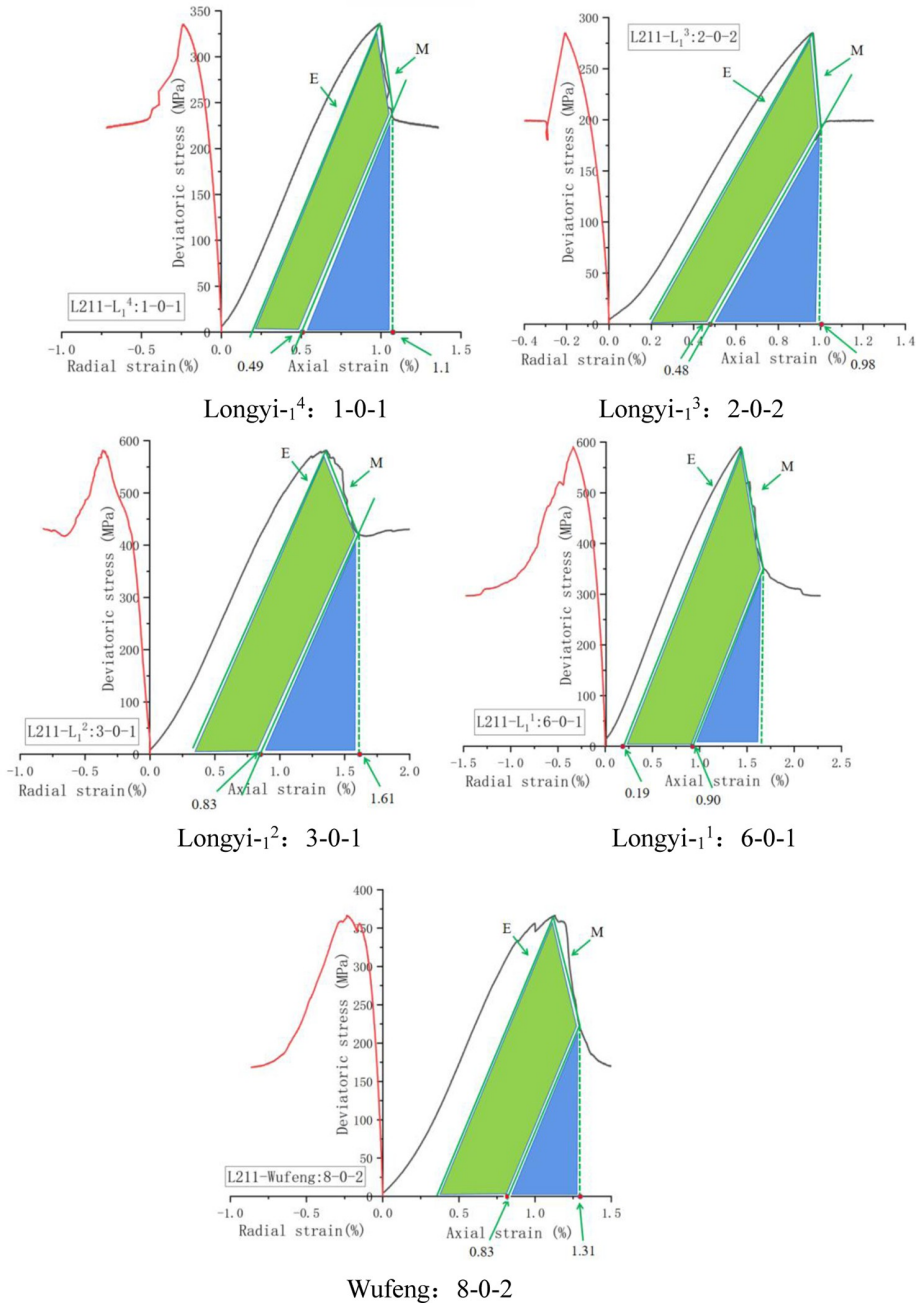
Brittleness evaluation of Lu211 well (energy dissipation method). Longyi-_1_^4^: 1-0-1, Longyi-_1_^3^: 2-0-2, Longyi-_1_^2^: 3-0-1, Longyi-_1_^1^: 6-0-1, Wufeng: 8-0-2.

Fig 8 shows the results obtained by describing the energy change in the stress-strain curve of the triaxial test according to the above formula:

Table 3 shows the evaluation results of the energy dissipation method.

**Table 3:** Summary of rock brittleness evaluation of Lu211 well (energy dissipation method).

Well	Position	Depth/m	Number	Confining pressure/MPa	M/GPa	E/GPa	BI_2_
Lu211	Longyi-_1_^4^	4900.92–4901.16	1-0-1	110	88.18	42.29	0.52
	Longyi-_1_^3^	4908.02–4908.30	2-0-2		67.14	36.23	0.46
	Longyi-_1_^2^	4917.64–4917.86	3-0-1		86.11	56.14	0.35
	Longyi-_1_^1^	4923.87–4924.05	6-0-1		80.00	47.19	0.41
	Wufeng	4929.39–4929.62	8-0-2		34.39	24.56	0.29

Note: Because the mineral composition of Baota Formation shale is too different from other rocks, the analysis of Baota Formation shale is not conducted in this section.

**Fig 9:**
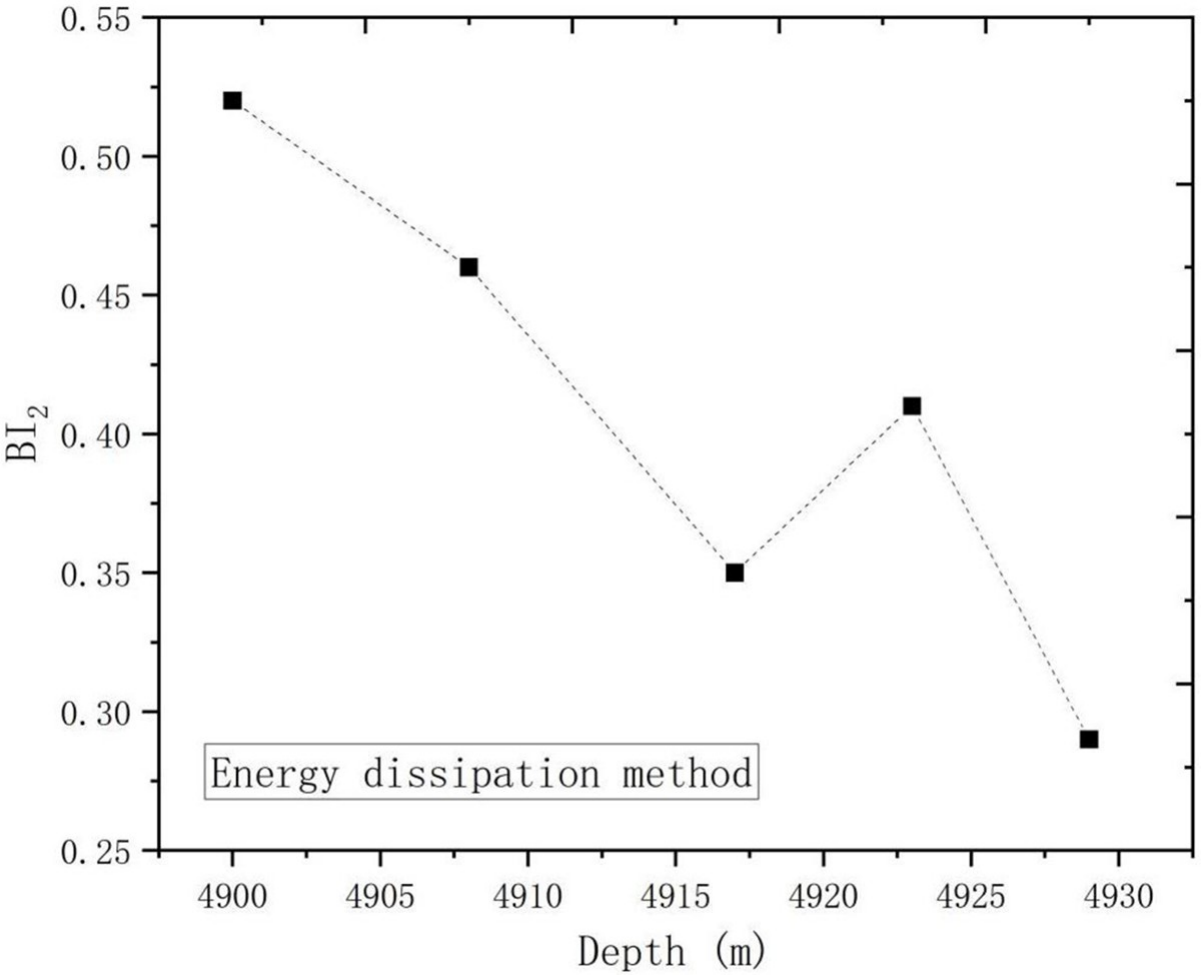
Brittleness index with depth (energy dissipation method).

Fig 9 shows the trend of brittleness index change with depth based on energy dissipation method. The figure illustrates that the brittleness index trends calculated by the energy dissipation method are comparable to those derived from the stress-strain method. Overall, the brittleness index tends to decrease as depth increases. The similarity in values obtained from both methods suggests that they are both viable for assessing the brittleness of deep shale.

### 3.3 Mineral composition method

Both stress strain method and energy dissipation method are based on the characteristics of the whole rock under the action of force, which indirectly reflect the brittleness characteristics of shale. The hardness, fracture characteristics, chemical composition and structure of the mineral components in the rock are the essential factors that determine the brittleness of the rock [21].

This section evaluates the brittleness characteristics of deep shale by analyzing the proportion of brittle minerals. As follows:

$$BI_{3}=\frac{W_{B}}{W_{total}}$$ 3-6

Where,

W_B_—Brittle mineral content;

W_total_—Total mineral composition.

As can be seen from Table 4, the contents of quartz and calcite are dramatically changed, so the influence of quartz and calcite is not considered. Therefore, the brittle minerals in the table include potassium feldspar, plagioclase, dolomite, pyrite and turbidite. The calculated brittleness index is shown in the table.

**Table 4:** Mineral components of Lu211 well.

Sample number	Mineral components (%)										BI_3_
	Quartz	Potassium feldspar	Plagioclase	Calcite	Dolomite	Pyrite	Laumontite	Clay mineral	W_B_	W_total_	
Longyi-_1_^4^	36.5	1.3	14.9	6.8	3.4	2.5	/	34.6	22.1	100	0.22
Longyi-_1_^3^	37.4	3.8	5.5	4.5	2.7	5	/	41.1	17		0.17
Longyi-_1_^2^	69.1	1.9	1.4	7.6	6.5	1.4	/	12.1	11.2		0.11
Longyi-_1_^1^	40.6	1	4.5	6.6	5.1	3.5	2.5	36.2	16.6		0.17
Wufeng	35.2	0.2	1.4	13.2	22.4	0.7	/	26.9	24.7		0.25
Baota	11.8	/	2	69.9	/	/	/	16.3	2		0.20

**Fig 10:**
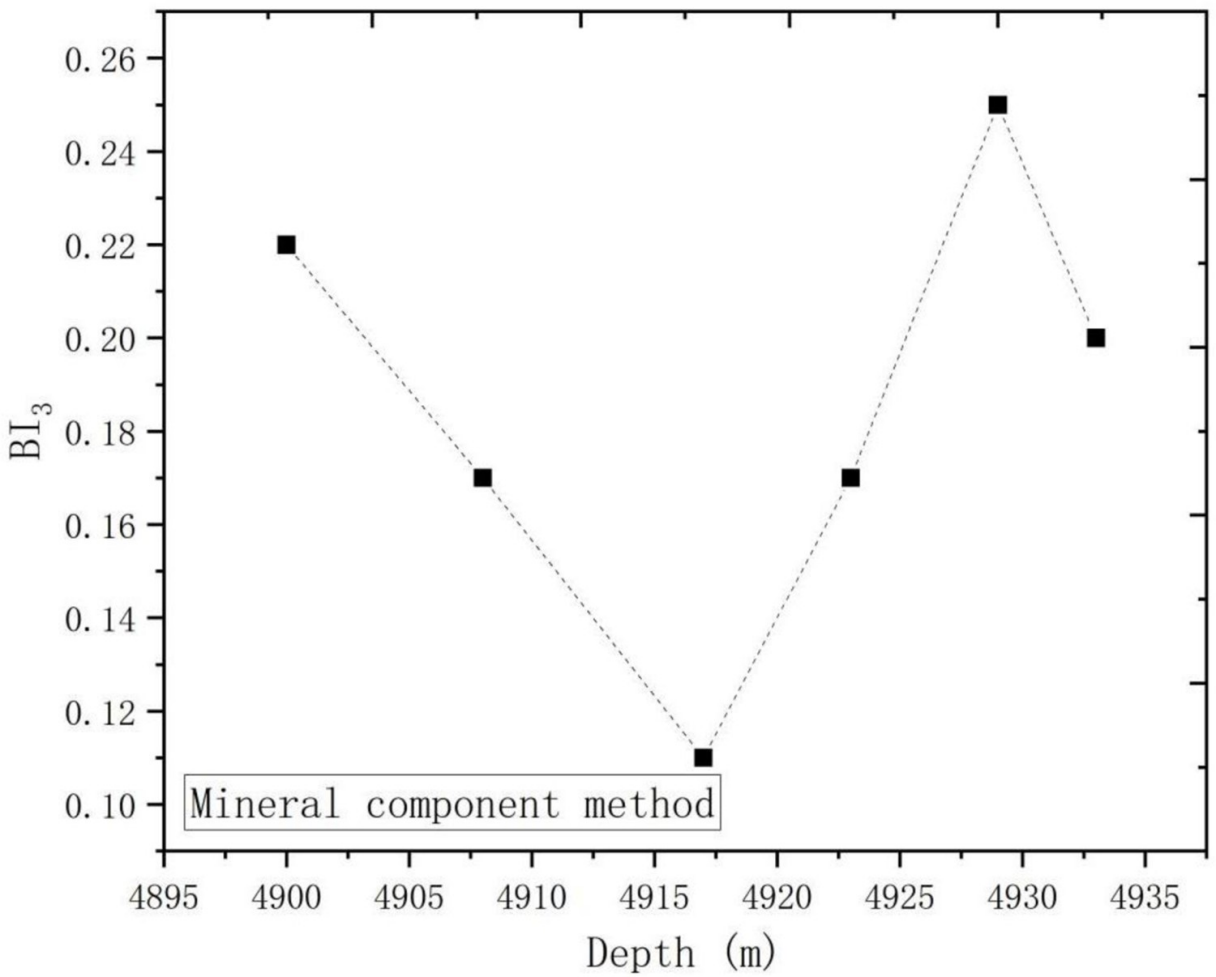
Brittleness index with depth (mineral component method).

According to Table 4, the trends of brittleness index with depth were plotted according to mineral component method, as shown in Fig 10. As can be seen from the figure, the brittleness index of deep shale calculated by mineral composition method showed a trend of first decreasing and then increasing with the increase of depth. This conclusion is different from the previous two methods, so the evaluation methods of deep shale need to be discussed comprehensively by various factors.

## 4 Discussions and expansions

**Fig 11:**
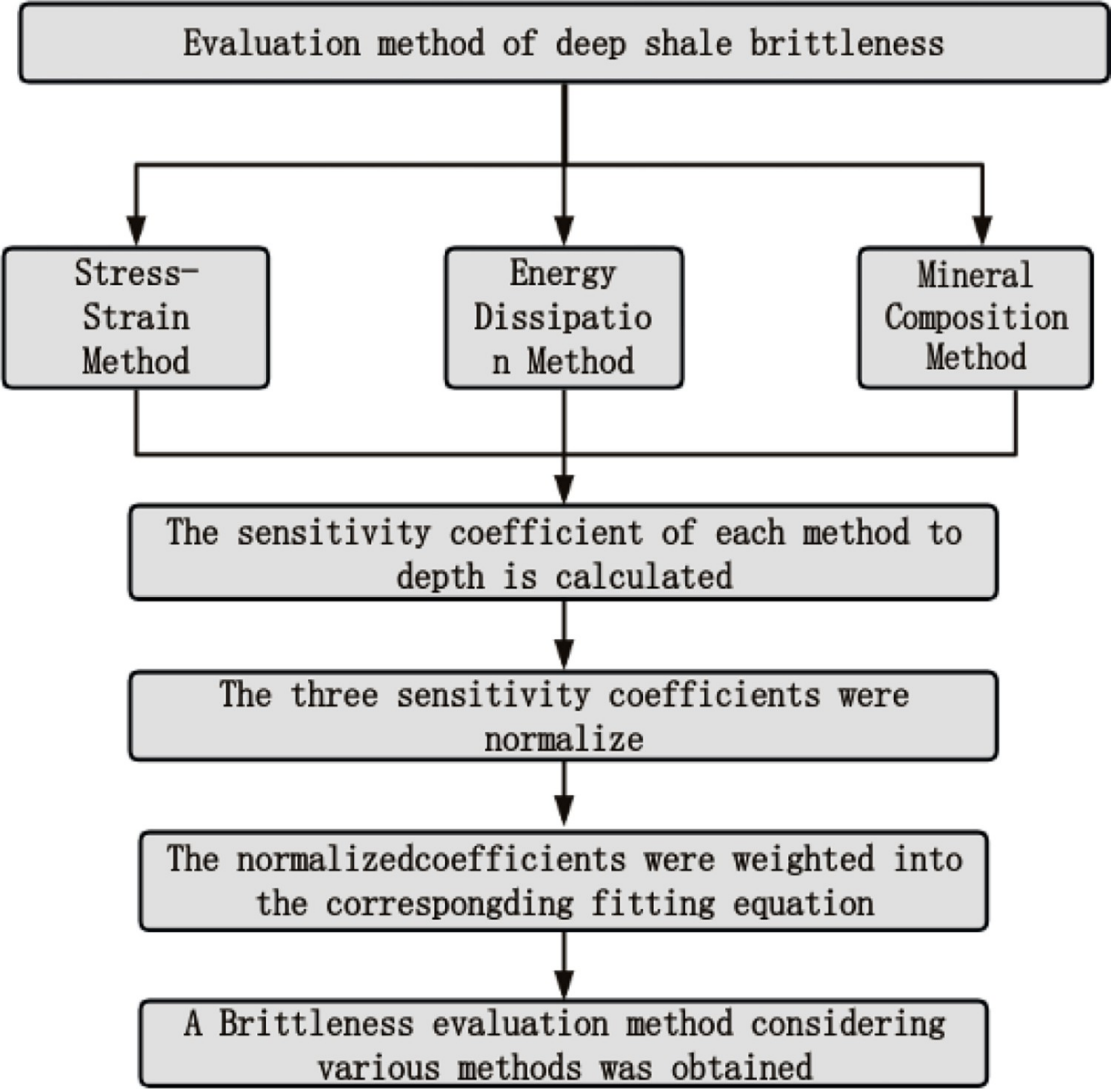
A brittleness evaluation method considering multiple methods.

**Fig 12:**
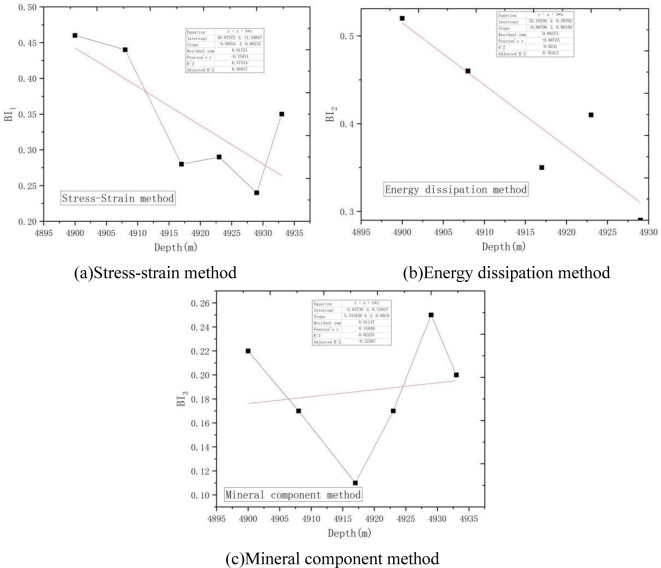
Curve fitting plot. (a)Stress-strain method, (b)Energy dissipation method, (c)Mineral component method.

From the previous study, we found that for the study of deep shale brittleness characteristics, it is necessary to synthesize the influence of all aspects. Here, a brittleness evaluation method considering various methods is obtained by using the weighted average method. The specific implementation method was shown in Fig 11:

Firstly, the depth-brittleness index relationship curves obtained by stress strain method, energy dissipation method and mineral composition method were fitted, as shown in Fig 12.

Sensitivity coefficients of stress-strain method, energy dissipation method and mineral composition method varying with depth were obtained from Fig 12, and the normalized results were shown in Table 5.

**Table 5:** Sensitivity coefficient normalization.

Brittleness evaluation method	Sensitivity coefficient	Normalized results
Stress-strain method	5.41×10^−3^	0.41
Energy dissipation method	7.06×10^−3^	0.54
Mineral composition method	0.57×10^−3^	0.04

Therefore, a brittleness evaluation method considering stress strain method, energy dissipation method and mineral composition method is obtained. As follows:

$$\begin{array}{l} BI=\alpha BI_{1}+\beta BI_{2}+\lambda BI_{3}\\ =0.41BI_{1}+0.54BI_{2}+0.04BI_{3} \end{array}$$ 4-1

**Fig 13:**
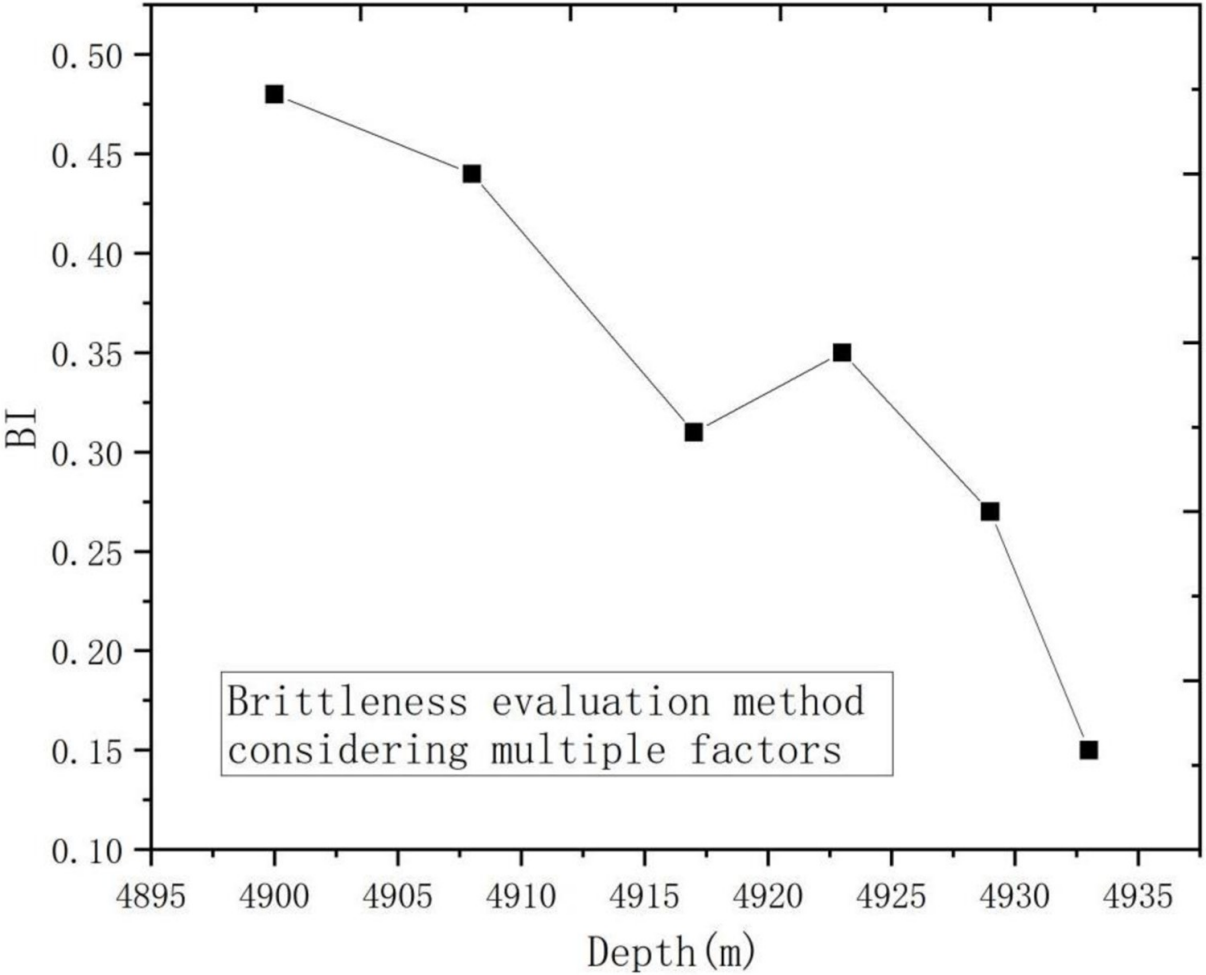
Brittleness evaluation method considering multiple factors.

According to the Formula, the results of brittleness evaluation method considering stress-strain method, energy dissipation method and mineral composition method were drawn, as shown in Fig 13.

As can be seen from Fig 13, the brittleness index of deep shale decreased with the increase of confining pressure. The results were similar to those obtained by stress-strain curve method and energy release method. However, due to the phase change of deep shale mineral composition, the results were different from those obtained based on mineral composition method. But in general, the results were consistent with common sense and can confirm the feasibility of the method.

## 5 Conclusions

This paper studies the brittleness characteristics of shale in the same well with the change of depth, taking each small layer of Lu211 well as the research object. The brittleness characteristics of deep shale were evaluated comprehensively from three aspects: mineral composition, stress-strain curve and energy dissipation. The following conclusions were drawn:

1. According to the brittleness evaluation method based on the stress-strain curve characteristics, the brittleness index of shale decreased with the increase of depth, but the brittleness index of the deepest Baota layer was higher. The reason is that the mineral components of the Baota layer undergo phase transformation and the brittleness of minerals increases rapidly.
2. When the brittleness evaluation method based on energy dissipation was used to evaluate the brittleness characteristics of deep shale, the law obtained is that with the increase of depth, the brittleness index of shale decreases, and the decreasing trend was relatively stable, without large abrupt change.
3. When the brittleness evaluation method based on mineral composition was used to study the change characteristics of deep shale brittleness with depth, the brittleness index was more discrete. This is because the diagenesis of deep shale is very different from that of shallow shale, so its mineral components are more discrete, so the brittleness index calculated by this method is more discrete.
4. Based on the principle of weighted average, a brittleness evaluation method considering various methods is designed, which can not only consider the influence of various factors, but also consider the influence weight of different methods on brittleness.

The study’s methodology highlights the complex and multi-dimensional aspects of shale brittleness, advocating for an integrative assessment that recognizes the synergistic effects of geological, mechanical, and materials science insights. By adopting a comprehensive approach, the research establishes a solid framework that deepens our comprehension of shale brittleness within the intricate context of deep geological strata. This sophisticated methodology not only advances our knowledge but also holds significant potential for refining geotechnical engineering designs and enhancing the strategic planning of resource extraction endeavors.
